# Dietary High Salt Intake Exacerbates SGK1-Mediated T Cell Pathogenicity in L-NAME/High Salt-Induced Hypertension

**DOI:** 10.3390/ijms25084402

**Published:** 2024-04-16

**Authors:** Dina Maaliki, Maha Itani, Hala Jarrah, Carla El-Mallah, Diana Ismail, Yara E. El Atie, Omar Obeid, Miran A. Jaffa, Hana A. Itani

**Affiliations:** 1 Department of Pharmacology and Toxicology, Faculty of Medicine, American University of Beirut, Beirut 1107, Lebanon; dsm07@mail.aub.edu (D.M.); mmi40@mail.aub.edu (M.I.); hhj15@mail.aub.edu (H.J.); dwi03@mail.aub.edu (D.I.); yee03@mail.aub.edu (Y.E.E.A.); 2 Department of Nutrition and Food Sciences, Faculty of Agricultural and Food Sciences, American University of Beirut, Beirut 1107, Lebanon; cae14@mail.aub.edu (C.E.-M.); oo01@aub.edu.lb (O.O.); 3 Epidemiology and Population Health Department, Faculty of Health Sciences, American University of Beirut, Beirut 1107, Lebanon; ms148@aub.edu.lb; 4 Division of Clinical Pharmacology, Department of Medicine, Vanderbilt University Medical Center, Nashville, TN 37232, USA

**Keywords:** inflammation, hypertension, SGK1, memory T cells, immunologic memory, T-lymphocytes

## Abstract

Sodium chloride (NaCl) activates Th17 and dendritic cells in hypertension by stimulating serum/glucocorticoid kinase 1 (SGK1), a sodium sensor. Memory T cells also play a role in hypertension by infiltrating target organs and releasing proinflammatory cytokines. We tested the hypothesis that the role of T cell SGK1 extends to memory T cells. We employed mice with a T cell deletion of SGK1, SGK1^fl/fl^ × tgCD4^cre^ mice, and used SGK1^fl/fl^ mice as controls. We treated the mice with L-NAME (0.5 mg/mL) for 2 weeks and allowed a 2-week washout interval, followed by a 3-week high-salt (HS) diet (4% NaCl). L-NAME/HS significantly increased blood pressure and memory T cell accumulation in the kidneys and bone marrow of SGK1^fl/fl^ mice compared to knockout mice on L-NAME/HS or groups on a normal diet (ND). SGK1^fl/fl^ mice exhibited increased albuminuria, renal fibrosis, and interferon-γ levels after L-NAME/HS treatment. Myography demonstrated endothelial dysfunction in the mesenteric arterioles of SGK1^fl/fl^ mice. Bone marrow memory T cells were adoptively transferred from either mouse strain after L-NAME/HS administration to recipient CD45.1 mice fed the HS diet for 3 weeks. Only the mice that received cells from SGK1^fl/fl^ donors exhibited increased blood pressure and renal memory T cell infiltration. Our data suggest a new therapeutic target for decreasing hypertension-specific memory T cells and protecting against hypertension.

## 1. Introduction

Hypertension is an enormous healthcare burden and a principal factor in global morbidity and mortality linked to myocardial infarction, stroke, and kidney disease. In some cases, hypertension occurs secondary to renal failure. However, in 95% of hypertensive cases, hypertension is considered primary and is associated with risk factors, such as obesity, aging, and/or salt consumption [1]. End-organ damage is the principal manifestation of hypertension and primarily affects the kidneys, heart, and vasculature. The American College of Cardiology/American Heart Association (ACC/AHA) released new guidelines in 2017 redefining hypertension as sustained blood pressure of ≥130/80 mmHg [2]. This classification places nearly half of the American population in the hypertensive category [3].

The blood pressure response to salt is not uniform among individuals. In that regard, the ACC/AHA defines salt sensitivity as “a physiological trait present in rodents and other mammals, including humans, in which the blood pressure of some members of the population exhibits changes parallel to changes in salt intake” [4]. Salt sensitivity affects approximately 50% of hypertensives and 25% of normotensives [4,5] and is an important risk factor for cardiovascular diseases (CVD) and mortality, independent of blood pressure elevation [6]. Recent findings indicate that sodium (Na^+^) accumulates in the interstitium and induces an inflammatory response. Reducing salt intake decreases blood pressure and cardiovascular risk [5,7,8,9,10]. Thus, the World Health Organization (WHO) recommends the consumption of no more than 5 g of salt/day [11]. 

Previously, research was mainly focused on hemodynamics to understand the pathophysiology of hypertension. Recent data however, have strongly pointed towards an essential role of the immune system in the harmful consequences of this disease. Hypertensive stimuli, including salt or angiotensin II (ANG II), activate components of the innate and adaptive immune systems, including damage-associated molecular patterns, the complement system, inflammasomes [12,13], dendritic cells (DCs), T cells, and macrophages [14,15,16]. High concentrations of extracellular salt induce lipid oxidation and the formation of isoketal adducts in DCs [17]. Activated DCs secrete inflammatory cytokines, such as interleukin (IL)-1, IL-6, and IL-23, which activate CD8^+^ and CD4^+^ T cells. Activated T cells migrate to the kidneys, vasculature, and other organs and induce extensive organ damage [18]. In the kidneys, T cells promote peritubular capillary apoptosis and cause Na^+^ water retention [19]. Importantly, an immunological basis for salt sensitivity was reported in Dahl salt-sensitive rats, with significant increases in renal T cell infiltration upon salt administration. 

Itani et al. uncovered a novel role of immunological memory in hypertension using two models of repeated hypertensive stimulation [20]: (1) high-dose ANG II infusion followed by low-dose ANG II infusion; (2) low-dose N(ω)-nitro-L-arginine methyl ester (L-NAME) treatment followed by high-salt (HS) exposure. They demonstrated that an initial hypertensive stimulus produces hypertension-specific effector memory T (TEM) cells that sensitize the host to a second mild challenge that would not have otherwise resulted in hypertension. L-NAME/HS induced immunological memory through cytokine-producing bone marrow-residing TEM cells that proliferated and trafficked to the kidneys upon repeated salt feeding. Most of these cells eventually die, leaving behind long-lived memory cells that reside in the bone marrow in a quiescent state for prolonged periods and are reactivated by repeated hypertensive stimulation. Once reactivated, TEMs expand and redistribute to the kidneys, thereby promoting renal damage. TEMs generally remain in the periphery and recirculate between the bone marrow and different organs. Another type of memory cells are central memory T(TCM) cells, which primarily reside in secondary lymphoid organs such as the spleen and lymph nodes [21]. TCM cells require a longer reactivation period than TEM cells but have a higher proliferative potential [22]. 

To study the mechanisms governing salt-sensitive HTN, the L-NAME/HS protocol was used. An important advantage of this model is that it induces immunological memory through repeated hypertensive stimuli without any surgical intervention; this mimicks the salt-sensitive HTN encountered in humans, making this model a particularly useful tool for studying inflammation. L-NAME is an important nitric oxide synthase (NOS) inhibitor, and inhibition of nitric oxide (NO) sets in motion a series of events that induce endothelial dysfunction and trigger an inflammatory response, which is key to developing salt sensitivity [23]. Reactive oxygen species (ROS) increase endothelial cell expression of adhesion molecules and chemokines [24], leading to monocyte transmigration and the activation of myeloid-derived DCs, which then activate T cells and prime HTN development [18,25]. Vascular dysfunction is another important pathogenic event resulting from low-dose L-NAME administration [26]. In this regard, rats treated with a low dose of L-NAME demonstrated subnormal vasodilation and elevated BP within 24 h of salt load, indicating that vascular dysfunction is responsible for initiating salt-sensitive HTN. After day 1, volume expansion in salt-sensitive mice was associated with increased NaCl cotransporter (NCC) levels, suggesting that volume expansion and sodium retention are necessary to maintain HTN. Importantly, impairment of renal sodium handling and increased NCC activity resulted from oxidative stress. Individuals with salt-sensitive HTN were reported to generate less NO than their salt-resistant hypertensive counterparts [27], and many genetic variations that modulate NOS activity are common in groups with a high prevalence of salt-sensitive HTN [4,28,29].

Serine/threonine protein kinase (SGK1), also known as “serum and glucocorticoid-regulated kinase”, is a recognized intracellular sensor of salt and a promoter of Na^+^ and volume retention in the kidney [30]. Interestingly, SGK1 was recently shown to be a key player in hypertension development in response to ANG II or salt. In antigen-presenting cells (APCs), SGK1 mediates the salt-induced expression and assembly of the epithelial sodium channel (ENaC) subunits ENaC-α and ENaC-γ, further increasing sodium influx in a positive feedback loop. Sodium influx promotes ROS formation and subsequent isolevuglandin (IsoLG) protein adducts, which trigger the inflammatory response [17,23]. Importantly, T cell-specific deletion of SGK1 attenuated HTN and renal and vascular T cell accumulation and injury after Ang II infusion or uninephrectomy followed by DOCA/salt administration [31]. Likewise, dendritic CD11c^+^ cell deletion of SGK1 attenuated renal T cell accumulation, endothelial dysfunction, and HTN during the HS feeding phase of L-NAME/HS administration [23]. Moreover, Dahl salt-sensitive rats exhibit increased sodium retention owing to augmented ENaC and SGK1 activity [32]. In addition to being activated through DCs, SGK1 directly promotes the differentiation of naïve T cells towards the T helper 17 (Th17) cell phenotype [33,34] and away from the protective T regulatory (Treg) cell phenotype [35], in response to elevated NaCl. Th17 cells, a subset of CD4^+^ T helper cells, produce IL-17A, which is essential for hypertension [36,37]. Studies demonstrated that mice lacking IL-17A are protected from hypertension and vascular dysfunction in response to ANG II [20,38,39]. The genetic ablation of SGK1 blunts proteinuria and renal fibrosis in response to elevated mineralocorticoid action [40,41]. In contrast, overactivation of SGK1 in a transgenic mouse model of unilateral nephrectomy and a 6-week treatment with deoxycorticosterone acetate and NaCl (DOCA/NaCl) produced a mild increase in glomerular filtration rate, enhanced albuminuria, and worsened glomerular hypertrophy and fibrosis [42]. To determine whether the phenotype associated with SGK1 activity extends to TEM cell formation under salt-sensitive conditions, we tested the hypothesis that T cell-specific SGK1 is necessary to form TEM cells and to develop salt-sensitive hypertension. 

## 2. Results

### 2.1. Specific Deletion of T Cell SGK1 Reduces L-NAME/HS-Induced Hypertension

**Figure 1:**
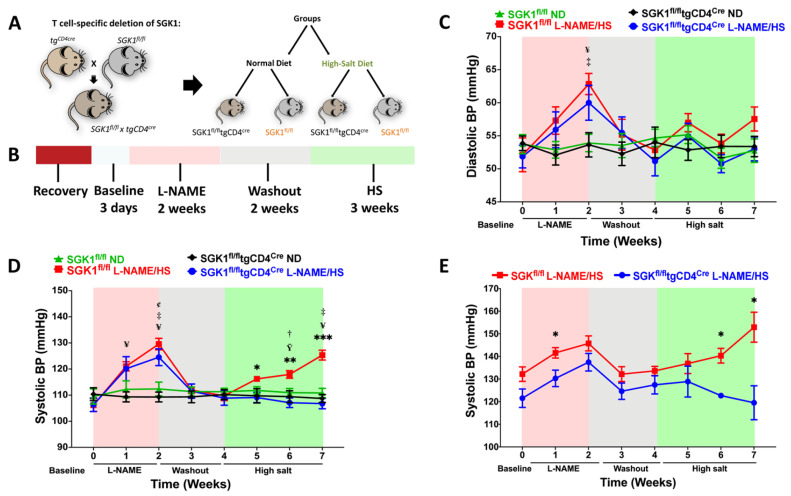
The loss of T cell SGK1 attenuates hypertension in response to L-NAME/HS administration. (**A**) SGK1^fl/fl^tgCD4^cre^ mice were produced by breeding SGK1^fl/fl^ mice with transgenic mice expressing Cre recombinase under the control of the CD4 promoter. (**B**) Protocol summary: SGK1^fl/fl^ mice and SGK1^fl/fl^tgCD4^cre^ mice received either an ND or L-NAME/HS; (**C**) diastolic and (**D**) systolic blood pressure were measured noninvasively using tail cuffs in conscious mice; (**E**) systolic blood pressure was invasively monitored in conscious mice through carotid radiotelemetry. (N = 12–15). Data are presented as mean ± standard error of the mean (SEM); *p*-values calculated by independent *t*-test are shown for data from two groups, and *p*-values calculated by multiple *t*-tests at each time point multiplied by several comparisons are shown for data from four groups. * *p* < 0.05, ** *p* < 0.01, *** *p* < 0.001 SGK1^fl/fl^ L-NAME/HS vs. SGK1^fl/fl^tgCD4^cre^ L-NAME/HS; † *p* < 0.05, ‡ *p* < 0.001 for SGK1^fl/fl^ L-NAME/HS vs. SGK1^fl/fl^ ND; Ÿ *p* < 0.05, ¥ *p* < 0.001 for SGK1^fl/fl^ L-NAME/HS vs. SGK1^fl/fl^tgCD4^cre^ ND; ¢ *p* < 0.001 for SGK1^fl/fl^tgCD4^cre^ L-NAME/HS vs. SGK1^fl/fl^tgCD4^cre^ ND. (L-NAME/HS, N(ω)-nitro-L-arginine methyl ester/high salt; SGK1, serine/threonine protein kinase 1; ND, normal diet).

We focused on identifying the role of SGK1 in promoting salt sensitivity in response to the L-NAME/HS protocol in mice with a T cell-specific deletion of SGK1. Therefore, we generated mice by using the Cre–loxP system. Breeding transgenic mice expressing Cre recombinase driven by the CD4 promoter (tgCD4^cre^) with SGK1^fl/fl^ mice resulted in a progeny featuring the T cell-specific knockout of SGK1. Because CD4 is expressed on all T cells during development, this method cross-deletes SGK1 in all T cells. SGK1^fl/fl^ mice that were not crossed with Cre were used as controls for the knockout mice (Figure 1A and Figure S1). This mouse model was previously characterized for ANG II-induced hypertension [31]. Blood pressure was measured using two methods: tail-cuff recording and telemetry. To induce recurrent episodes of hypertension, the mice underwent an initial treatment with the NOS inhibitor L-NAME (0.5 mg/mL) in drinking water for two weeks. This was followed by a two-week washout period, after which they were placed on a high-salt (HS) diet containing 4% NaCl for three weeks (Figure 1B). Systolic and diastolic blood pressures were measured biweekly via a tail cuff (Figure 1C,D). No significant differences were detected in diastolic blood pressure, measured by the tail cuff, except during the L-NAME phase of the protocol in mice exposed to L-NAME/HS compared to mice that received ND (Figure 1C). Systolic blood pressure in both groups increased with L-NAME initiation and returned to baseline after L-NAME discontinuation. No differences in the baseline systolic blood pressure were observed. On the other hand, during the HS phase of the protocol, SGK1^fl/fl^ mice exhibited increased systolic blood pressure in response to the HS diet, in contrast to SGK1^fl/fl^ × tgCD4^cre^ mice, which were protected from developing a hypertensive response (Figure 1D,E). A separate group of mice underwent a radiotelemetry transmitter implantation for systolic blood measurement and reported similar results (Figure 1E). The heart rates were similar among the four groups at the end of the protocol (Supplementary Figure S2). 

### 2.2. SGK1 Mediates Renal Inflammation and Promotes Renal Injury

**Figure 2:**
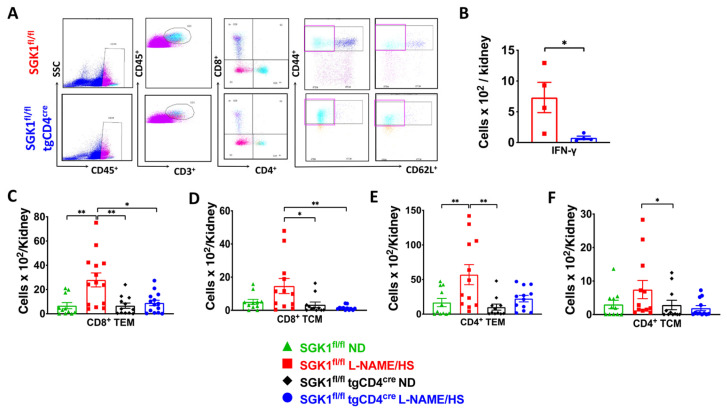
SGK1 mediates renal inflammation in response to L-NAME/HS. (**A**) Representative flow cytometric gating strategy of single-cell tissue suspensions after L-NAME/HS treatment for total leukocytes (CD45^+^ expression), total T lymphocytes (CD3^+^), CD8^+^ and CD4^+^ T cells, CD8^+^ and CD4^+^CD44^hi^/CD62L^lo^ (TEM cells), and CD44^hi^/CD62L^hi^ (TCM cells). SSC-A indicates a side scatter area, and FSC-A indicates a forward scatter area. Purple boxes indicate CD4^+^ and CD8^+^ TEM cells; (**B**) intracellular staining to assess renal-infiltrating CD44^hi^ memory T cells following L-NAME/HS treatment (N = 4); (**C**–**F**) summary flow cytometric quantification of absolute numbers of kidney-infiltrating CD8^+^ and CD4^+^ (TEM and TCM) cells in SGK1^fl/fl^ and SGK1^fl/fl^tgCD4^cre^ mice in response to L-NAME/HS vs. ND (N = 11–14). Data are expressed as mean ± SEM. *p*-values calculated by independent *t*-test are shown, and *p*-values calculated by 2-way ANOVA or non-parametric test are shown for data from four groups. * *p* < 0.05, ** *p* < 0.01.

TEM cells are activated in response to repeated hypertensive stimuli and produce cytokines, including IL-17 and interferon γ (IFNγ), which promote renal injury and end-organ damage. Previous studies indicated that the number of CD4^+^CD62L^lo^ and CD8^+^CD62L^lo^ TEM cells increases in the kidney in response to treatment with L-NAME followed by an HS diet [20]. Flow cytometry was performed on single-cell suspensions of the kidneys from both groups exposed to the L-NAME/HS protocol. The gating strategy shown in Figure 2A identified total leukocytes (CD45^+^ cells), total T lymphocytes (CD45^+^CD3^+^ cells), and subsets of T lymphocytes, including CD4^+^ and CD8^+^ T cells. Representative flow cytometry dot plots and a gating strategy showed an increase in the number of CD8^+^ TEM (CD8^+^CD44^hi^CD62L^lo^) and TCM (CD8^+^CD44^hi^CD62L^hi^) cells in the kidneys of SGK1^fl/fl^ mice on L-NAME/HS but not in other groups (Figure 2C,D). The differences in the number of total kidney T lymphocytes (CD45^+^CD3^+^ cells), CD4^+^ T lymphocytes, and CD8^+^ T lymphocytes across the four groups are presented in Supplementary Figure S2. Intracellular staining revealed elevated production of IFN-γ in renal CD44^hi^ memory T cells of hypertensive SGK1^fl/fl^ mice compared with SGK1^fl/fl^ × tgCD4^cre^ mice, as shown in Figure 2B.

**Figure 3:**
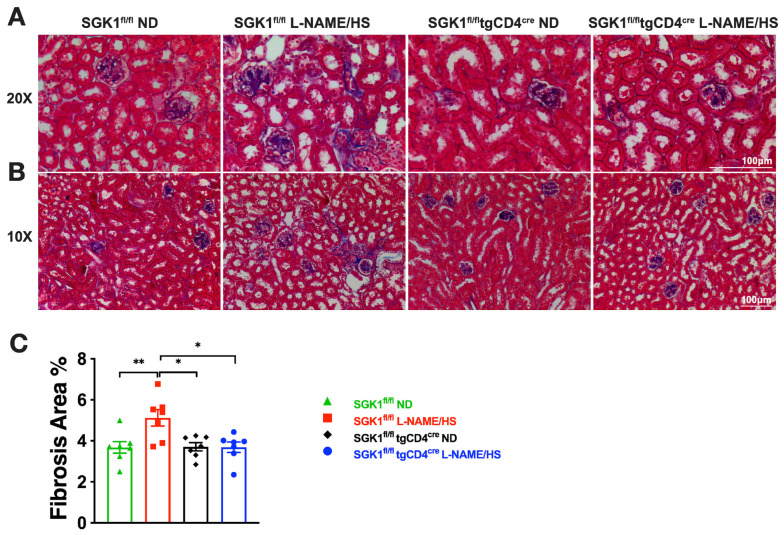
SGK1 promotes renal fibrosis in response to L-NAME/HS. (**A**,**B**) Masson’s trichrome staining of the kidney to detect renal fibrosis at (**A**) 20× magnification and (**B**) 10× magnification (N = 7); (**C**) data are expressed as mean ± SEM, *p*-values calculated by 2-way ANOVA are shown. * *p* < 0.05, ** *p* < 0.01.

**Figure 4:**
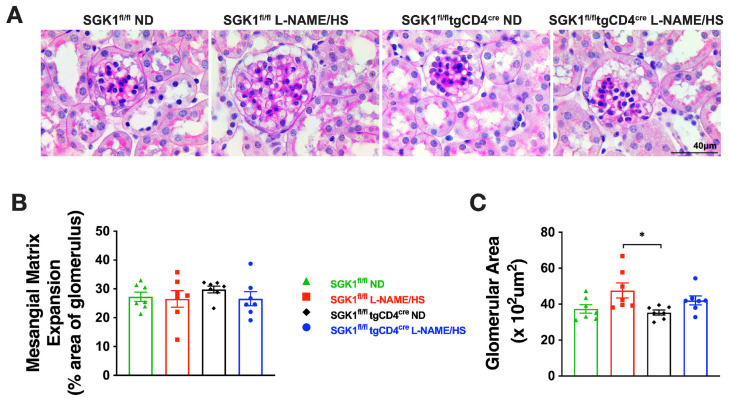
SGK1 promotes glomerular hypertrophy in response to L-NAME/HS in SGK1^fl/fl^ mice. (**A**) PAS staining of the kidney at 40× magnification to determine (**B**) glomerular and (**C**) mesangial matrix areas (N = 7). Data are expressed as mean ± SEM, and *p*-values calculated by 2-way ANOVA are shown. * *p* < 0.05.

**Figure 5:**
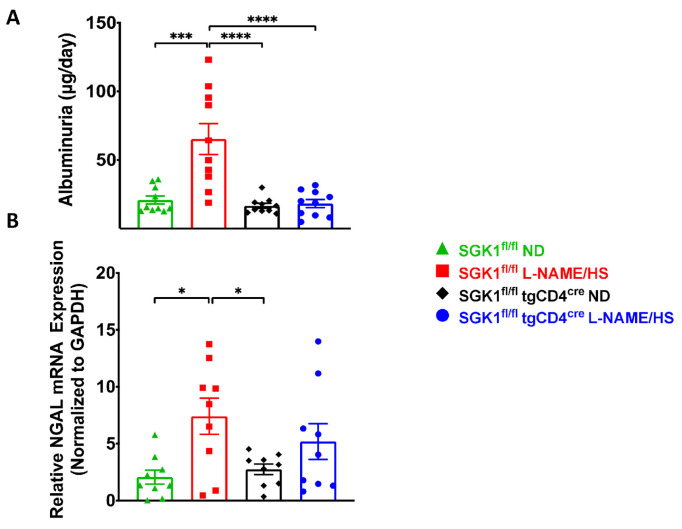
SGK1 promotes renal injury in response to L-NAME/HS. (**A**) Glomerular injury was assessed by quantifying 24 h urinary albumin in SGK1^fl/fl^ and SGK1^fl/fl^tgCD4^cre^ mice at the end of the L-NAME/HS protocol (N = 10); (**B**) tubular injury was examined with NGAL mRNA expression using RT-qPCR (N = 9). Data are presented as mean ± SEM. *p*-values were derived using 2-way ANOVA and are displayed for datasets encompassing the four groups. * *p* < 0.05, *** *p* < 0.001, **** *p* < 0.0001.

To evaluate renal injury, we performed Masson’s trichrome and Periodic acid–Schiff (PAS) staining to assess fibrosis and glomerular changes respectively, and measured urinary albumin and renal neutrophil gelatinase-associated lipocalin (NGAL) to assess glomerular and tubular injury respectively. L-NAME/HS increased kidney fibrosis (Figure 3A–C) in SGK1^fl/fl^ mice. PAS staining of kidney sections demonstrated a significantly increased glomerular area in SGK1^fl/fl^ mice on L-NAME/HS compared to SGK1^fl/fl^ × tgCD4^cre^ mice on an ND (Figure 4A,B). There was no difference in the mesangial area among the four groups (Figure 4A–C). Albumin was measured from 24 h urine samples collected at the end of the L-NAME/HS protocol, and demonstrated significant increases in SGK1^fl/fl^ mice (Figure 5A). We also found a statistically significant elevation in renal NGAL mRNA expression in the groups fed L-NAME/HS compared to the groups that received the control diet at the end of the treatment protocol. While no difference was noted between the two groups subjected to L-NAME/HS, a trend for higher NGAL levels was observed in the SGK1^fl/fl^ mice (Figure 5B).

### 2.3. T Cell SGK1 Deficiency Protects against Vascular Inflammation and Injury

**Figure 6:**
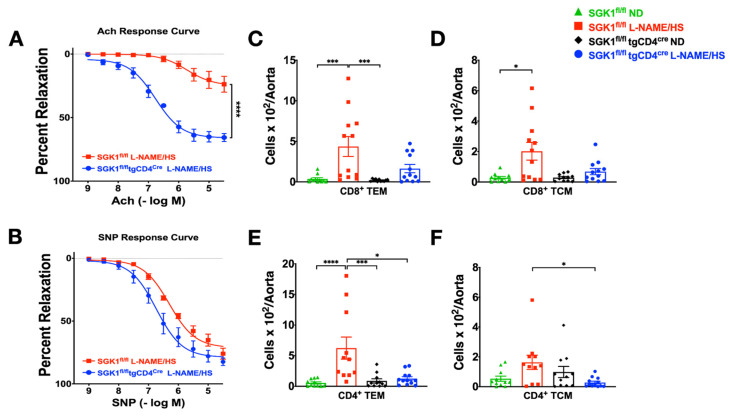
T cell SGK1 deficiency protects against vascular inflammation and function. (**A**) Endothelium-dependent relaxation in response to increasing doses of Ach and (**B**) endothelium-independent relaxation in response to increasing doses of SNP were measured after L-NAME/HS administration; (**C**–**F**) flow cytometric quantification of total CD8^+^ and CD4^+^ (TEM and TCM) cells in SGK1^fl/fl^ mice and SGK1^fl/fl^ × tgCD4^cre^ mice on L-NAME/HS vs. ND in the aorta (N = 11–12). Data are expressed as mean ± SEM. *p*-values calculated by independent *t*-test are shown for data from two groups, and *p*-values calculated by 2-way ANOVA or non-parametric tests are shown for data from four groups. * *p* < 0.05, *** *p* < 0.001, **** *p* < 0.0001.

Studies has demonstrated that SGK1 in T cells mediates vascular dysfunction in response to hypertensive stimuli [31]. However, the mesenteric vessels from SGK1^fl/fl^ mice exposed to the L-NAME/HS protocol demonstrated impaired vascular relaxation in response to ACh but not to SNP. In contrast, SGK1^fl/fl^ × tgCD4^cre^ mice were protected from vascular dysfunction (Figure 6A,B). This indicated that T cell SGK1 plays a role in endothelium-dependent relaxation. Previous studies emphasized the role of the perivascular infiltration of leukocytes in the development of vascular inflammation and stiffness. Flow cytometry of single-cell suspensions of the thoracic aorta demonstrated no significant increase in the number of CD8^+^ TEM and TCM cells in the aorta of SGK1^fl/fl^ mice compared to SGK1^fl/fl^ × tgCD4^cre^ mice on L-NAME/HS (Figure 6C,D). However, there was a strong trend towards an increased number of TEM cells in SGK1^fl/fl^ mice on L-NAME/HS. Compared with mice fed the ND, SGK1^fl/fl^ mice fed L-NAME/HS demonstrated a significant increase in the number of CD4^+^ and CD8^+^ TEM cells. In addition, the number of CD4^+^ TCM cells was significantly higher in SGK1^fl/fl^ mice than in SGK1^fl/fl^ × tgCD4^cre^ mice on L-NAME/HS (Figure 6F).

### 2.4. SGK1 Is Necessary for Bone Marrow Memory T Cells Accumulation

**Figure 7:**
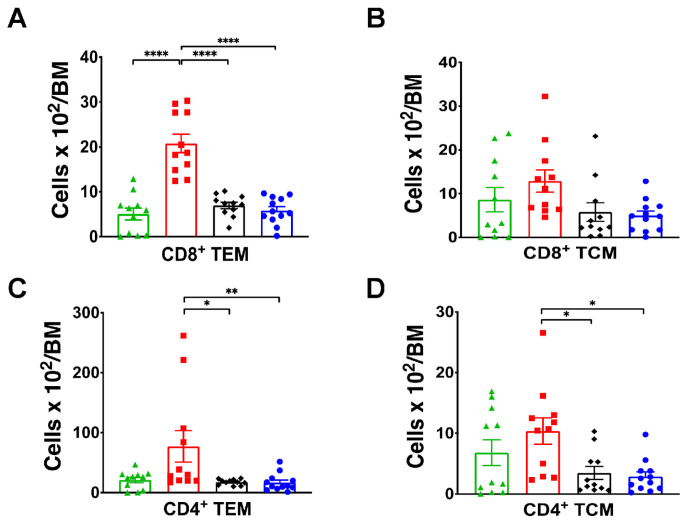
Bone marrow TEMs exhibit salt sensitivity. (**A**–**D**) Summary data of the absolute numbers of CD8^+^ and CD4^+^ TEM and TCM cells in SGK1^fl/fl^ and SGK1^fl/fl^tgCD4^cre^ mice following the administration of L-NAME/HS in the bone marrow (N = 11–12). * *p* < 0.05, ** *p* < 0.01, **** *p* < 0.0001.

We hypothesized that T cell specific SGK1 is necessary to form memory T cells in the bone marrow. We previously demonstrated that exposure to L-NAME/HS causes a 1.8-fold increase in CD4^+^ T cells and a 3-fold increase in CD8^+^ T cells in the bone marrow [20]. Flow cytometry demonstrated that CD4^+^ TCM and CD8^+^ and CD4^+^ TEM cells were significantly lower in the SGK1^fl/fl^ x tgCD4^cre^ mice on HS and in mice on control diet compared to SGK1^fl/fl^ on L-NAME/HS (Figure 7).

### 2.5. Adoptive Transfer of Bone Marrow TEM Cells from SGK1^fl/fl^ Mice Promotes Salt Sensitivity

**Figure 8:**
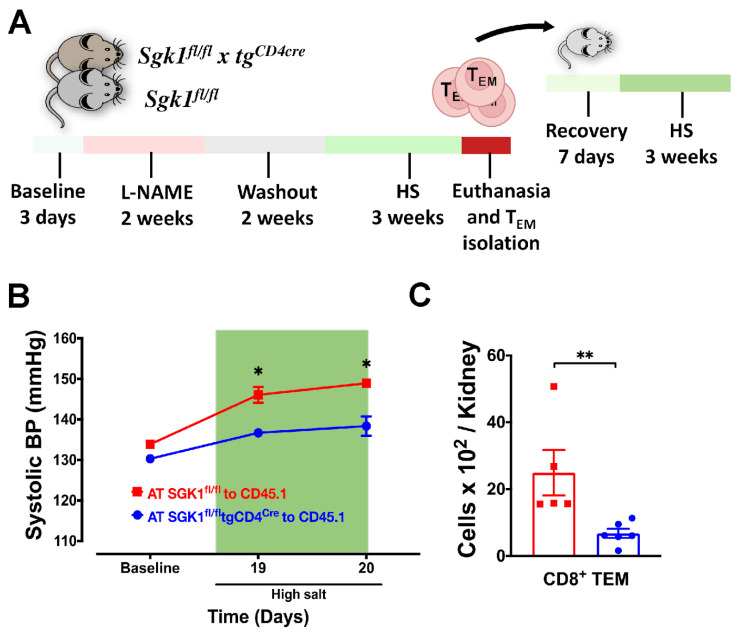
Adoptive transfer of bone marrow TEM cells promotes salt sensitivity (**A**) Experimental paradigm: bone marrow TEM cells from donor SGK1^fl/fl^ or SGK1^fl/fl^tgCD4^cre^ mice fed L-NAME/HS were adoptively transferred to recipient CD45.1 mice. Recipient mice were then fed an HS diet for three weeks; (**B**) blood pressure radiotelemetry of recipient mice throughout the L-NAME/HS protocol; (**C**) renal CD8^+^ TEM cells were quantified in recipient mice following HS feeding (N = 5–6). Data are expressed as mean ± SEM. *p*-values for the difference in blood pressure between the two groups calculated by independent *t*-test or Mann–Whitney U test are shown; *p*-values calculated by 2-way ANOVA or non-parametric tests are shown for data from four groups. * *p* < 0.05, ** *p* < 0.001.

**Figure 9:**
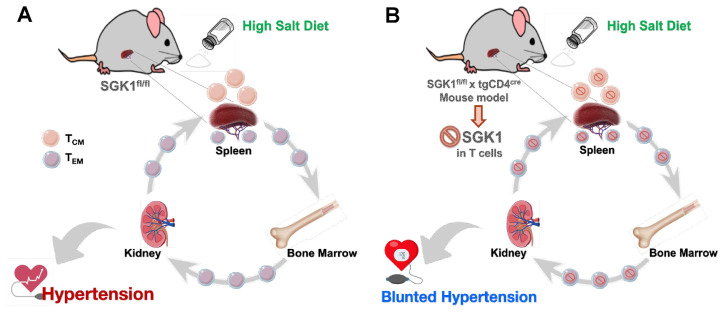
Schematic illustrating how memory T cells contribute to salt sensitivity. (**A**,**B**) T cell-specific deletion of SGK1 decreased memory T cell number, salt sensitivity, and blood pressure elevation following L-NAME/HS exposure.

Bone marrow TEM cells from CD45.2 donor SGK1^fl/fl^ × tgCD4^cre^ mice or SGK1^fl/fl^ mice at the end of the L-NAME/HS treatment were sorted and adoptively transferred to recipient CD45.1 mice (Figure 8A). Following recovery, the mice were fed an HS diet for three weeks. Radiotelemetry measurements demonstrated that the mice that received bone marrow TEM cells from SGK1^fl/fl^ × tgCD4^cre^ mice were protected from developing a hypertensive response, in contrast to the mice that received TEM cells from SGK1^fl/fl^ mice (Figure 8B). We also measured CD8^+^ TEM cell accumulation in the kidneys of the recipient mice; the CD8^+^ TEM cell levels were significantly higher in the mice with TEM cells adoptively transferred from SGK1^fl/fl^ mice than in those with TEM cells transferred from SGK1^fl/fl^ × tgCD4^cre^ mice (Figure 8C). A schematic representation of the role of SGK1 in salt-sensitive hypertension is shown in Figure 9.

## 3. Discussion

This study emphasizes the role of the immune system in hypertension and related end-organ damage. The L-NAME/HS protocol was used to mimic salt-sensitive hypertension. L-NAME inhibits NO production, which mimics endothelial NO loss in many diseases such as hypertension and diabetes. NO also attenuates lipid peroxidation and the formation of lipid peroxidation products known as isoketals. Consequently, L-NAME promotes the formation of highly immunogenic isoketals in DCs [18]. Activated DCs stimulate T cell activation and hypertension development [18]. 

SGK1 modulates Na^+^ transport in renal epithelial cells by upregulating NCC and ENaC [30]. Interestingly, SGK1 in T cells and DCs is critical for developing salt-dependent and salt-independent hypertension [18,31]. Mice lacking SGK1 in T cells or DCs are protected from hypertension, renal and vascular T cell accumulation and injury in response to salt stress [17,31]. SGK1 can also directly activate T cells, without help from DCs. For example, SGK1 directly stimulates the differentiation of naïve T cells into Th17 cells [33,34,36]. In addition, SGK1 was found to be a key player in mineralocorticoid/salt-induced hypertension [40,41,42]. Here, we aimed to study the role of SGK1 in promoting salt-sensitive hypertension by modulating TEM cells. We demonstrated that mice lacking SGK1 in T cells exhibited blunted hypertension in response to L-NAME/HS. To our knowledge, this is the first study to examine the role of SGK in promoting immunological memory.

The kidney is essential in hypertension immunology [43,44,45]. Renal denervation prevents the accumulation of T cells in the kidneys and the subsequent inflammatory response [43]. Moreover, TEM cells accumulate in the kidney and release inflammatory cytokines such as IFN-γ and IL-17A, which are responsible for renal damage [20]. Our research indicates the importance of SGK1 in the accumulation of CD8^+^ TEM and TCM cells in the kidney. Mice lacking SGK1 in T cells did not accumulate memory cells or IFN-γ in the kidney in response to HS. Salt-sensitive hypertension leads to widespread renal fibrosis, which has important clinical implications in the progression to chronic glomerulosclerosis and end-stage renal failure [46,47,48]. In agreement with this, we demonstrated that SGK1^fl/fl^ × tgCD4^cre^ mice were protected from fibrotic kidney damage. Albuminuria is an important marker of glomerular damage. It was previously shown that SGK1^fl/fl^ × tgCD4^cre^ mice were protected from renal damage after four weeks of ANG II infusion [31]. In line with this, albuminuria increased in wild-type mice exposed to L-NAME/HS [20]. Compared to SGK1^fl/fl^ mice on L-NAME/HS, SGK1^fl/fl^ × tgCD4^cre^ mice on either diet were protected from albuminuria. Significant increases in the glomerular area were observed in SGK1^fl/fl^ mice on L-NAME/HS compared to SGK1^fl/fl^ × tgCD4^cre^ mice on ND, but not between the two groups on the L-NAME/HS diet. Recently, it was shown that transgenic mice with increased SGK1 activity displayed increased glomerular hypertrophy and fibrosis upon exposure to DOCA/NaCl for 6 weeks and unilateral nephrectomy [42]. Interestingly, renal effects were observed without an increase in blood pressure or glomerular filtration rate, indicating that an increased SGK1 function could serve as a risk factor for the development of hypertension-independent kidney damage. Therefore, the role of SGK1 in glomerular hypertrophy has not been completely elucidated. Our results may be due to the combined effect of HS and the SGK1 deletion or to the fact that more time is needed to observe the full extent of glomerular alterations. Regarding the assessment of tubular injury, we demonstrated increased NGAL expression in the kidneys of both groups on L-NAME/HS compared to the groups on the ND, consistent with previous data from our group [20]. However, no significant differences were observed between the two L-NAME/HS groups. Thus, it is possible that SGK1 in T cells does not participate in tubular injury. Importantly, biological factors related to the C57Bl/6 genetic background could, at least in part, account for the observed effects on kidney function. The C57Bl/6 mouse genetic background is the most used model for gene knockout studies. However, it is known to possess observable resistance to hypertension-induced renal damage. C57Bl/6 mice demonstrated significant resistance to glomerulosclerosis, interstitial fibrosis, and albuminuria induced by DOCA/salt compared to other mouse strains [49]. Resistance to renal injury was also observed in C57Bl/6 mice with other models of hypertension, such as ANG II infusion, protein overload, and 5/6 nephrectomy [50,51,52]. Therefore, inter-strain differences should be taken into consideration.

Hypertension is associated with T cell infiltration into perivascular fat [53]. Moreover, salt-sensitive individuals cannot reduce their systemic vascular resistance after salt loading [54]. SGK1 plays an essential role in vascular damage, and the deletion of SGK1 in T cells protects vessels from increased vascular inflammation in response to ANG II [31]. We demonstrated that SGK1^fl/fl^ × tgCD4^cre^ mice were protected from vascular endothelial dysfunction. Interestingly, the increase in T cell accumulation in the aortas of SGK1^fl/fl^ mice exposed to ANG II was driven by CD4^+^ and double-negative T cells, and no significant differences were observed in CD8^+^ T cells. Likewise, our study only found significant differences in CD4^+^ TEM and TCM cell infiltration in the aortas of SGK1^fl/fl^ mice compared with SGK1^fl/fl^ × tgCD4^cre^ mice after L-NAME/HS. No differences with in CD8^+^ TEM and TCM cell accumulation between the groups on L-NAME/HS. This is in line with previous research that did not observe an increase in the number of aortic memory T cells after L-NAME/HS exposure [20]. At present, it is unclear why this was the case. However, evidence indicates that additional exposure to HS may eventually lead to incremental increases in the number of memory T cells. When wild-type mice that underwent the L-NAME/HS protocol were re-exposed to a second 3-week HS challenge, they exhibited further increases in the number of CD4^+^ and CD8^+^ TEM cells in the kidney compared to when they were exposed to the first challenge [20]. Therefore, it is possible that more time or repeated challenges are needed to observe significant memory T cell increases in the aorta.

Accumulating data indicate that the bone marrow plays a crucial role in the pathogenesis of HTN. Studies indicate that the adoptive transfer of bone marrow T cells from hypertensive mice is sufficient to promote hypertension development in naïve normotensive mice [55,56]. As previously mentioned, TEM cells are reactivated in response to stimuli [20,57]. Exposure to the L-NAME/HS protocol was previously shown to induce significant increases in the number of CD4^+^ and CD8^+^ T cells in the bone marrow [20]. In this study, flow cytometry of single-cell suspensions from the bone marrow also demonstrated significant increases in the number of both CD4^+^ TCM cells (CD44^hi^/CD62L^hi^) and CD8^+^ and CD4^+^ TEM cells (CD44^hi^/CD62L^lo^) in SGK1^fl/fl^ mice compared to SGK1^fl/fl^ × tgCD4^cre^ mice, indicating that SGK1 plays a key role in the induction of immunological memory. In addition, TEM cells adoptively transferred from the bone marrow of SGK1^fl/fl^ mice exposed to the L-NAME/HS protocol showed increased CD8^+^ TEM cell accumulation in the kidneys in response to HS. This is in line with previous data on the adoptive transfer of SGK1-deficient DCs [23] and TEM cells [20]. Van Beusecum et al. exposed splenic CD11c^+^ cells from SGK1^fl/fl^ and SGK1^CD11c^KO mice to HS culture media for 48 h and transferred these cells to wild-type mice, which received a 4-week infusion of a subpressor dose of ANG II [23]. The authors demonstrated that the wild-type mice that received DCs lacking SGK1 were protected from a hypertensive response compared to those that received SGK1^fl/fl^ APCs. Similarly, Itani et al. demonstrated that adoptively transferred bone marrow TEM cells from hypertensive mice into recipient CD45.1 mice homed to the bone marrow and spleen and then expanded upon salt feeding [20]. 

It is important to note that some of the observed renal changes may occur independently of elevated blood pressure. Indeed, Na^+^ can accumulate in hyperosmolar states in physiological tissues without accompanying increases in water, challenging the previous assumption that the Na^+^ concentration in the interstitial space is like that in the plasma [7,58,59]. This is important because elevated levels of local extracellular salt can stimulate the key elements of the immune response. In cell culture and experimental models of autoimmunity, salt was shown to exert local effects on T cell repertoire, dendritic cell function, and inflammatory cytokine release [14,17,33,34,36,60]. IL-17A and IFN-γ directly impair the kidney function by modulating the activity of multiple renal transporters, including NCC, sodium potassium chloride cotransporter (NKCC), and sodium hydrogen exchanger-3 (NHE3) [36,39,61,62,63]. They are also major players in the local vascular production of ROS and vascular stiffening [39,64,65,66,67]. Indeed, IL-17 was shown to modulate eNOS synthase activity [64]. In addition to IL-17A and IFN-γ, renal SGK1 mediates the renal function through the local regulation of sodium and potassium homeostasis [30,68]. Interestingly, SGK1 was shown to be a key player in kidney damage from mineralocorticoids through a mechanism that is independent of blood pressure elevation [42]. The mechanisms thought to be responsible for this include the upregulation of SGK1 in renal podocytes, which increases glomerular ROS levels [69], or SGK1-mediated upregulation of mesangial cell intercellular adhesion molecule (ICAM-1) and connective tissue growth factor (CTGF) expression, which promotes renal fibrosis [70]. Moreover, sympathetic activation orchestrates inflammation. In line with this, immune cells express adrenergic receptors, which have been directly implicated in inflammatory mechanisms [43,71]. A unilateral renal denervation mouse model allowed for a uniform pressure in both kidneys. Significantly less renal immune cell infiltration was observed in the denervated kidney than in the innervated kidney, further indicating the important contributions of hypertension-independent sympathetic activation.

Our study has some limitations. Transgenic SGK1^fl/fl^ × tgCD4^cre^ mice have a deletion of SGK in T cells that express CD4 at any stage during development, including a subset of γδ T cells and double-positive CD4^+^ CD8^+^ T cells, some of which eventually mature into single-positive CD4^+^ and CD8^+^ T cells [72,73]. Previous evidence showed that in mice with ANG-II-induced HTN, γδ T cells are an important source of IL-17A in the kidneys and vasculature [37]. The role of SGK1 in γδ T cells in salt-sensitive hypertension remains unclear. However, we observed significant differences in hypertension, memory T cell formation, and organ dysfunction in mice, indicating a significant role of SGK1 in salt-sensitive hypertension. In addition, animal [74,75] and human studies [76,77,78] demonstrated important sex-specific differences in salt sensitivity, which are largely attributed to the influence of sex hormones, aldosterone [75,77,79], and mineralocorticoid receptor function [74,80,81]. Male mice were used in this study; thus, based on our results, future studies will be dedicated to understanding sex differences in salt-sensitive hypertension. Another key limitation relates to the nature of the transgenic mouse models. Importantly, transgenic models are highly effective for understanding disease pathogenesis and investigating therapeutic measures. However, due to physiological differences between humans and mice, it is important to note that most human diseases cannot be fully replicated in mice, even in situations where most disease parameters are observed. Another potential limitation of our study is the possible confounding effect of age on immune function and hypertension. Salt sensitivity, blood pressure, and kidney function worsen with advancing age [82,83,84,85,86]. To date, studies demonstrated considerable variation in the age at which hypertension develops in C57BL/6 wild-type mice. De Moudt et al. demonstrated that blood pressure did not change from baseline until 12 months of age [87]. In another study, it was found that blood pressure progressively began to increase as early as at 12 weeks of age but did not significantly change until 28 weeks of age [88]. In that regard, the Klotho protein is an anti-aging protein that is produced in the kidney and excreted in a soluble form into the circulation. Renal and circulating Klotho levels decline with age and CKD [89,90,91] and Klotho deficiency is correlated with salt sensitivity in hypertensive patients [91,92,93,94]. Moreover, 18-week-old mice were protected from salt-induced increases in blood pressure, which was attributed to Klotho sufficiency, compared to mice aged 66 weeks, which had significantly lower Klotho levels [94]. Considering the above data and the comparable ages of our mice, increased age may not have heavily influenced our results. Moreover, because the mice were age-matched throughout the protocol, we can expect the potential confounding effect of age to be uniform across groups. However, it is difficult to confirm this with certainty, especially without signs of overt age-related kidney damage or an investigation of age-related markers.

An exciting area for future development is the identification of neoantigens by T cell receptor (TCR) sequencing. This would provide critical information on TCR repertoire, transcript length frequencies, non-Gaussian distributions, and T cell clonality. Trott et al. found dominant TCR transcript lengths in Vβ3, 8.1, and 17 families of renal CD8^+^ cells in ANG II-treated mice [95], indicating neoantigen formation and T cell activation in the kidney. The future identification of peptide sequence patterns that stimulate immune responses will enhance the development of medications and vaccines against self- and neoantigens. In conclusion, our findings suggest a novel role of SGK1 in accumulating and expanding memory cells in salt-sensitive hypertension. Additional research is required to understand the role of the immune system in hypertension and elucidate the molecular signals involved in inflammation-induced hypertension. 

## 4. Materials and Methods

### 4.1. Animals and Study Design

The study animals were housed at the American University of Beirut Animal Care Facility, and the animal experiments were confirmed by the facility’s Animal Ethics Committee to adhere to established ethical guidelines for animal research in compliance with the IACUC Guidelines for the Ethical Use of Animals in Research. The mice were housed at 25 °C under a regular 12 h cycle of light and darkness, had free access to water, and were fed a consistent Teklad diet. At the end of the study, the animals were euthanized using carbon dioxide. SGK1^fl/fl^ mice were bred with tgCD4^Cre^ transgenic mice, which resulted in the deletion of SGK1 in T cells. tgCD4^cre^ mice were obtained from Jackson Laboratories and maintained on the C57BL/6 background. SGK1^fl/fl^ mice were purchased from Aniko Naray-Fejes-Toth (Dartmouth College, Hanover, NH, USA) and bred as previously described [96]. These two groups of mice (SGK1^fl/fl^ and SGK1^fl/fl^ × tgCD4^cre^ mice) were further divided to receive either a normal diet (ND) (0.4% NaCl) or an L-NAME/HS diet. The L-NAME/HS protocol was performed as previously described [7]. Male mice aged 10–12 weeks received L-NAME (0.5 mg/mL, Cayman, Ann Arbor, MI, USA) in drinking water for two weeks. Then, L-NAME was discontinued, and a washout phase with regular drinking water for two weeks was implemented. Mice were fed a 3-week HS diet (4% NaCl; Harlan, KI, USA). Norlander et al. validated the efficiency of SGK1 deletion in SGK1^fl/fl^tgCD4^cre^ mice [31]. Similarly, we validated SGK1 deletion in T cells (Supplementary Figure S1).

### 4.2. Blood Pressure Measurement

Non-invasive tail-cuff method: the BP-2000 Blood Pressure Analysis System™ was utilized for non-invasive measurements of systolic and diastolic blood pressure, along with heart rate measurements. The mice were acclimatized to the apparatus for one week prior to the initial measurements. Each session involved 30 cycles of blood pressure readings, with 10 preliminary and 20 recorded cycles. The sessions were conducted twice weekly throughout the study, with data processed using BP-2000 Analysis Software, 2024 Visitech, Inc., Drammen, Norway.

Telemetry: blood pressure was monitored invasively using radiotelemetry (Stellar Telemetry, TSE Systems, Midland, MI, USA) following established protocols [97]. The mice recovered 10 days after surgery before exposure to L-NAME/HS.

### 4.3. Flow Cytometry Analysis of Immune Cells

Single-cell suspensions from the bone marrow, kidney, and thoracic aorta were prepared according to established protocols [20]. The kidneys were mechanically dissociated in a gentleMACS C tube using a gentleMACS dissociator system (Miltenyi, Bergisch, Gladbach, Germany). Subsequently, the cell suspensions were incubated for 20 min at 37 °C with gentle rotation in a solution containing 2 mg/mL of collagenase D (Roche Diagnostics, Mannheim, Germany) and 100 µg/mL of DNase I (Roche Diagnostics) in RPMI 1640 medium supplemented with 5% FBS. The resulting kidney homogenates were filtered through a 70 µm cell strainer and subjected to Percoll gradient centrifugation (GE Healthcare, Upsala, Sweeden). The cells collected from the Percoll interface were rinsed with cold phosphate-buffered saline (PBS). For bone marrow cell isolation, the tibias and femurs were centrifuged at 17,750× *g* for 5 min and then passed through 40 µm strainers with RPMI medium. The RBC lysis buffer (eBioscience, Waltham, MA, USA) was used to eliminate the blood cells. Subsequently, cell counts were performed, with 500,000 cells allocated for adoptive transfer, and one million for flow cytometry analysis [20]. Thoracic aortas were excised, cleared of perivascular adipose tissue, minced, and enzymatically digested at 37 °C for 30 min using a digestion cocktail of collagenase types A and B (Roche Diagnostics, Mannheim, Germany) in RPMI 1640, with rotation in a hybridization oven. After digestion, the cells were centrifuged at 800× *g* for 5 min, resuspended in PBS, and strained through a 70 µm cell strainer (Falcon, BD Biosciences, Franklin Lakes, NJ, USA) to remove undigested tissue and debris [45].

Extracellular staining: The prepared single-cell suspensions were washed and labeled with LIVE/DEAD^®^ Fixable Violet Dead Cell Stain (Invitrogen, Eugene, OR, USA). The staining process involved Brilliant Violet 510 (BV510)-conjugated anti-CD45 antibody, peridinin chlorophyll protein–cyanine-5.5 (PerCP-Cy5.5)-conjugated anti-CD3 antibody, allophycocyanin–cyanin-7 (APC-Cy7)-conjugated anti-CD4 antibody, phycoerythrin–cyanin-7 (PE-Cy7)-conjugated anti-CD8a antibody, APC-conjugated anti-CD44 antibody, PE-conjugated anti-CD62L antibody, and FITC-conjugated anti-F4/80 antibody (BioLegend, San Diego, CA, USA). Before the analysis, each sample, excluding those from the bone marrow, was supplemented with 50 µL of 1,2,3 count eBeadsTM (Invitrogen, Van Allen Way, Carlsbad, CA, USA). The samples were processed using a BD FACS Aria SORP cell sorter, and the data were analyzed with FlowJo software V10 (Tree Star Inc., San Carlos, CA, USA), setting gates based on fluorescence minus one (FMO) control [45]. 

Intracellular staining: Fixation and permeabilization were achieved with 4% paraformaldehyde and 0.1% Triton X-100 for intracellular staining. In total, 1 × 10^6^ kidney cells were resuspended in 200 μL of 4% paraformaldehyde dissolved in 1× PBS and incubated for 20 min at room temperature. The cells were washed twice with 1× PBS and incubated for 15 min at a density of 1 × 10^6^ cells in 1 mL of 0.1% Triton X-100. A PE–dazzle-conjugated anti-IFN-γ antibody was used.

The results were normalized using bead counts and expressed as the number of cells per kidney or aorta. All lymphocyte subpopulations (CD4^+^ and CD8^+^) were quantified using the CD45^+^CD3^+^gate.

### 4.4. Mesenteric Vascular Reactivity

Second-order mouse mesenteric arterioles were isolated from the perivascular fat. Then, 2 mm segments were used to perform isometric tension studies. A small-vessel horizontal wire myograph (DMT model 620M, Danish Myo Technology, Hinnerup, Denmark) was used. Tone was recorded for each vessel using LabChart Pro 8 (ADInstruments Ltd., Dunedin, New Zealand) with a physiological salt solution containing 130 mM NaCl, 4.7 mM KCl, 1.2 mM MgSO_4_, 1.2 mM KH_2_PO_4_, 25 mM NaHCO_3_, 5 mM glucose, and 1.6 mM CaCl2. The vessels were equilibrated for 20 min at 37 °C. A passive circumference–tension curve was generated for each vessel to determine the optimum passive tension to simulate an in vivo transmural pressure of 100 mmHg, as previously described, with modifications [98]. After normalization, vessel integrity was assessed using KCl (60 mM). The vessels were pre-constricted with phenylephrine (PE; 1 μM) and exposed to increasing concentrations of acetylcholine (ACh) or sodium nitroprusside (SNP) to test endothelium-dependent and endothelium-independent vascular relaxation, respectively. 

### 4.5. Measurements of Renal Injury Markers

#### Neutrophil Gelatinase-Associated Lipocalin (NGAL) mRNA Quantification

Kidney samples were lysed and homogenized in TRIzol Reagent (Invitrogen, Waltham, MA, USA) according to the manufacturer’s protocol and then subjected to phenol–chloroform extraction. RNA yield and quality were determined using a DeNovix spectrophotometer (DeNovix DS-11 FX Spectrophotometer, Wilmington, DE, USA). The qualitative conversion of the RNA extracts into single-stranded cDNA was performed using a high-capacity cDNA reverse transcription (RT) kit (Applied Biosystems, Foster City, CA, USA). 

To determine the cDNA yield from the reverse transcription reaction, qPCR was performed using the SensiFAST SYBR^®^ No-ROX Mix kit (Meridian Biosciences, Cincinnati, OH, USA) for each sample in duplicate, following the manufacturer’s instructions. The PCR plate was loaded into a CFX384 Touch real-time PCR detection system and incubated for 2 min at 95 °C, followed by 5 s at 95 °C (40 cycles), 60 s at 60.3 °C, and 15 s at 72 °C. Fold changes in the expression of the gene of interest were calculated using the Ct method and were normalized to the expression of GAPDH. 

### 4.6. Kidney Histology

After perfusion of the mice with PBS, the kidneys were fixed in 10% neutral buffered formalin, routinely processed, paraffin-embedded, and cut into 5 μm sections. These sections were subsequently stained with Masson’s trichrome stain and analyzed by a blinded observer. All steps, in addition to dehydration, clearing, and coverslipping, were performed using a Leica Bond-Max IHC autostainer. The slides were then deparaffinized. Heat-induced antigen retrieval was performed using the Epitope Retrieval 2 solution for 10 min. Masson’s trichrome staining was performed using a Gemini autostainer. Kidney section imaging was performed at 20× magnification and 10× magnification. Fibrosis was analyzed as the area of fibrosis using ImageJ software, V2.14.0/1.54f. Periodic acid–Schiff staining (PAS) was used to examine the glomerular area. Images were collected at 40× magnification, and the glomerular area was traced and measured using ImageJ software. A 40× objective was used to obtain images of mesangial expansion. The images were analyzed using ImageJ software. The mesangial expansion area was calculated as the ratio of the mesangial matrix area to the total glomerular area and expressed as a percentage, as explained by Rangan and Tesch [99].

### 4.7. T Cells Adoptive Transfer Studies

TEM cells were isolated from the bone marrow of SGK1^fl/fl^ × tgCD4^cre^ and SGK1^fl/fl^ mice (CD 45.2 donors, Jackson Laboratories, Bar Harbor, ME, USA) and sorted using specific antibodies against CD44 CD62L using a cell sorter. In total, 500,000 cells were adoptively transferred to wild-type CD 45.1 recipient mice by tail vein injection [20]. After seven days of recovery, the mice were fed an HS diet for 3 weeks. Blood pressure was measured and recorded at baseline and biweekly during each week of the 3-week HS phase. Euthanasia was performed using CO_2_. 

## Data Availability

All data for this study will be made available upon reasonable request to the corresponding author.

## Supplementary Materials

The following supporting information can be downloaded at: https://www.mdpi.com/article/10.3390/ijms25084402/s1.

## References

[1] Carretero O.A., Oparil S. (2000). Essential hypertension: Part II: Treatment. Circulation.

[2] Whelton P.K., Carey R.M., Aronow W.S., Casey D.E., Collins K.J., Dennison Himmelfarb C., DePalma S.M., Gidding S., Jamerson K.A., Jones D.W. (2018). 2017 ACC/AHA/AAPA/ABC/ACPM/AGS/APhA/ASH/ASPC/NMA/PCNA Guideline for the Prevention, Detection, Evaluation, and Management of High Blood Pressure in Adults: Executive Summary: A Report of the American College of Cardiology/American Heart Association Task Force on Clinical Practice Guidelines. Hypertension.

[3] Ostchega Y., Fryar C.D., Nwankwo T., Nguyen D.T. (2020). Hypertension Prevalence among Adults Aged 18 and over: United States, 2017–2018.

[4] Elijovich F., Weinberger M.H., Anderson C.A., Appel L.J., Bursztyn M., Cook N.R., Dart R.A., Newton-Cheh C.H., Sacks F.M., Laffer C.L. (2016). Salt sensitivity of blood pressure: A scientific statement from the American Heart Association. Hypertension.

[5] He F.J., Li J., Macgregor G.A. (2013). Effect of longer-term modest salt reduction on blood pressure: Cochrane systematic review and meta-analysis of randomised trials. BMJ.

[6] Weinberger M.H., Fineberg N.S., Fineberg S.E., Weinberger M. (2001). Salt sensitivity, pulse pressure, and death in normal and hypertensive humans. Hypertension.

[7] Machnik A., Neuhofer W., Jantsch J., Dahlmann A., Tammela T., Machura K., Park J.K., Beck F.X., Müller D.N., Derer W. (2009). Macrophages regulate salt-dependent volume and blood pressure by a vascular endothelial growth factor-C-dependent buffering mechanism. Nat. Med..

[8] Dahl L., Love R. (1954). Relation of sodium chloride intake to essential hypertension in humans. Fed. Proc..

[9] Stamler J., Elliott P., Kesteloot H., Nichols R., Claeys G., Dyer A.R., Stamler R. (1996). Inverse relation of dietary protein markers with blood pressure. Findings for 10,020 men and women in the INTERSALT Study. Circulation.

[10] Obarzanek E., Proschan M.A., Vollmer W.M., Moore T.J., Sacks F.M., Appel L.J., Svetkey L.P., Most-Windhauser M.M., Cutler J.A. (2003). Individual blood pressure responses to changes in salt intake: Results from the DASH-Sodium trial. Hypertension.

[11] WHO (2012). Guideline: Sodium Intake for Adults and Children.

[12] Wenzel P., Knorr M., Kossmann S., Stratmann J., Hausding M., Schuhmacher S., Karbach S.H., Schwenk M., Yogev N., Schulz E. (2011). Lysozyme M-positive monocytes mediate angiotensin II-induced arterial hypertension and vascular dysfunction. Circulation.

[13] Wenzel U., Turner J.E., Krebs C., Kurts C., Harrison D.G., Ehmke H. (2016). Immune Mechanisms in Arterial Hypertension. J. Am. Soc. Nephrol. JASN.

[14] Binger K.J., Gebhardt M., Heinig M., Rintisch C., Schroeder A., Neuhofer W., Hilgers K., Manzel A., Schwartz C., Kleinewietfeld M. (2015). High salt reduces the activation of IL-4- and IL-13-stimulated macrophages. J. Clin. Investig..

[15] Harrison D.G., Guzik T.J., Lob H.E., Madhur M.S., Marvar P.J., Thabet S.R., Vinh A., Weyand C.M. (2011). Inflammation, immunity, and hypertension. Hypertension.

[16] Jantsch J., Schatz V., Friedrich D., Schröder A., Kopp C., Siegert I., Maronna A., Wendelborn D., Linz P., Binger K.J. (2015). Cutaneous Na+ storage strengthens the antimicrobial barrier function of the skin and boosts macrophage-driven host defense. Cell Metab..

[17] Barbaro N.R., Foss J.D., Kryshtal D.O., Tsyba N., Kumaresan S., Xiao L., Mernaugh R.L., Itani H.A., Loperena R., Chen W. (2017). Dendritic Cell Amiloride-Sensitive Channels Mediate Sodium-Induced Inflammation and Hypertension. Cell Rep..

[18] Kirabo A., Fontana V., de Faria A.P., Loperena R., Galindo C.L., Wu J., Bikineyeva A.T., Dikalov S., Xiao L., Chen W. (2014). DC isoketal-modified proteins activate T cells and promote hypertension. J. Clin. Investig..

[19] McMaster W.G., Kirabo A., Madhur M.S., Harrison D.G. (2015). Inflammation, immunity, and hypertensive end-organ damage. Circ. Res..

[20] Itani H.A., Xiao L., Saleh M.A., Wu J., Pilkinton M.A., Dale B.L., Barbaro N.R., Foss J.D., Kirabo A., Montaniel K.R. (2016). CD70 Exacerbates Blood Pressure Elevation and Renal Damage in Response to Repeated Hypertensive Stimuli. Circ. Res..

[21] Samji T., Khanna K.M. (2017). Understanding memory CD8^+^ T cells. Immunol. Lett..

[22] Sallusto F., Geginat J., Lanzavecchia A. (2004). Central memory and effector memory T cell subsets: Function, generation, and maintenance. Annu. Rev. Immunol..

[23] Van Beusecum J.P., Barbaro N.R., McDowell Z., Aden L.A., Xiao L., Pandey A.K., Itani H.A., Himmel L.E., Harrison D.G., Kirabo A. (2019). High Salt Activates CD11c^+^ Antigen-Presenting Cells via SGK (Serum Glucocorticoid Kinase) 1 to Promote Renal Inflammation and Salt-Sensitive Hypertension. Hypertension.

[24] Le Brocq M., Leslie S.J., Milliken P., Megson I.L. (2008). Endothelial dysfunction: From molecular mechanisms to measurement, clinical implications, and therapeutic opportunities. Antioxid. Redox Signal..

[25] Randolph G.J., Beaulieu S., Lebecque S., Steinman R.M., Muller W.A. (1998). Differentiation of monocytes into dendritic cells in a model of transendothelial trafficking. Science.

[26] Wang C., Kawakami-Mori F., Kang L., Ayuzawa N., Ogura S., Koid S.S., Reheman L., Yeerbolati A., Liu B., Yatomi Y. (2020). Low-dose L-NAME induces salt sensitivity associated with sustained increased blood volume and sodium-chloride cotransporter activity in rodents. Kidney Int..

[27] Ghiadoni L., Virdis A., Taddei S., Gonzales J., Salazar J., Andersen L., Duranti P., Salvetti A. (1997). Defective nitric oxide-pathway in salt-sensitive essential hypertensive patients. Am. J. Hypertens..

[28] Sverdlov A.L., Ngo D.T., Chan W.P., Chirkov Y.Y., Horowitz J.D. (2014). Aging of the nitric oxide system: Are we as old as our NO?. J. Am. Heart Assoc..

[29] Svetkey L.P., McKeown S.P., Wilson A.F. (1996). Heritability of salt sensitivity in black Americans. Hypertension.

[30] Ellison D.H. (2013). Ubiquitylation and the pathogenesis of hypertension. J. Clin. Investig..

[31] Norlander A.E., Saleh M.A., Pandey A.K., Itani H.A., Wu J., Xiao L., Kang J., Dale B.L., Goleva S.B., Laroumanie F. (2017). A salt-sensing kinase in T lymphocytes, SGK1, drives hypertension and hypertensive end-organ damage. JCI Insight.

[32] Aoi W., Niisato N., Sawabe Y., Miyazaki H., Tokuda S., Nishio K., Yoshikawa T., Marunaka Y. (2007). Abnormal expression of ENaC and SGK1 mRNA induced by dietary sodium in Dahl salt-sensitively hypertensive rats. Cell Biol. Int..

[33] Kleinewietfeld M., Manzel A., Titze J., Kvakan H., Yosef N., Linker R.A., Muller D.N., Hafler D.A. (2013). Sodium chloride drives autoimmune disease by the induction of pathogenic TH17 cells. Nature.

[34] Wu C., Yosef N., Thalhamer T., Zhu C., Xiao S., Kishi Y., Regev A., Kuchroo V.K. (2013). Induction of pathogenic TH17 cells by inducible salt-sensing kinase SGK1. Nature.

[35] Hernandez A.L., Kitz A., Wu C., Lowther D.E., Rodriguez D.M., Vudattu N., Deng S., Herold K.C., Kuchroo V.K., Kleinewietfeld M. (2015). Sodium chloride inhibits the suppressive function of FOXP3+ regulatory T cells. J. Clin. Investig..

[36] Norlander A.E., Saleh M.A., Kamat N.V., Ko B., Gnecco J., Zhu L., Dale B.L., Iwakura Y., Hoover R.S., McDonough A.A. (2016). Interleukin-17A regulates renal sodium transporters and renal injury in angiotensin II–induced hypertension. Hypertension.

[37] Saleh M.A., Norlander A.E., Madhur M.S. (2016). Inhibition of Interleukin 17-A but not Interleukin-17F Signaling Lowers Blood Pressure and Reduces End-organ Inflammation in Angiotensin II-induced Hypertension. JACC Basic. Transl. Sci..

[38] Madhur M.S., Lob H.E., McCann L.A., Iwakura Y., Blinder Y., Guzik T.J., Harrison D.G. (2010). Interleukin 17 promotes angiotensin II-induced hypertension and vascular dysfunction. Hypertension.

[39] Kamat N.V., Thabet S.R., Xiao L., Saleh M.A., Kirabo A., Madhur M.S., Delpire E., Harrison D.G., McDonough A.A. (2015). Renal transporter activation during angiotensin-II hypertension is blunted in interferon-γ−/− and interleukin-17A−/− mice. Hypertension.

[40] Vallon V., Huang D.Y., Grahammer F., Wyatt A.W., Osswald H., Wulff P., Kuhl D., Lang F. (2005). SGK1 as a determinant of kidney function and salt intake in response to mineralocorticoid excess. Am. J. Physiol.-Regul. Integr. Comp. Physiol..

[41] Artunc F., Amann K., Nasir O., Friedrich B., Sandulache D., Jahovic N., Risler T., Vallon V., Wulff P., Kuhl D. (2006). Blunted DOCA/high salt induced albuminuria and renal tubulointerstitial damage in gene-targeted mice lacking SGK1. J. Mol. Med..

[42] Sierra-Ramos C., Velazquez-Garcia S., Keskus A.G., Vastola-Mascolo A., Rodríguez-Rodríguez A.E., Luis-Lima S., Hernández G., Navarro-González J.F., Porrini E., Konu O. (2021). Increased SGK1 activity potentiates mineralocorticoid/NaCl-induced kidney injury. Am. J. Physiol. Renal Physiol..

[43] Xiao L., Kirabo A., Wu J., Saleh M.A., Zhu L., Wang F., Takahashi T., Loperena R., Foss J.D., Mernaugh R.L. (2015). Renal denervation prevents immune cell activation and renal inflammation in angiotensin II–induced hypertension. Circ. Res..

[44] Maaliki D., Itani M.M., Itani H.A. (2022). Pathophysiology and genetics of salt-sensitive hypertension. Front. Physiol..

[45] Itani M.M., Jarrah H., Maaliki D., Radwan Z., Farhat R., Itani H.A. (2022). Sphingosine 1 phosphate promotes hypertension specific memory T cell trafficking in response to repeated hypertensive challenges. Front. Physiol..

[46] Berger R.C., Vassallo P.F., Crajoinas Rde O., Oliveira M.L., Martins F.L., Nogueira B.V., Motta-Santos D., Araújo I.B., Forechi L., Girardi A.C. (2015). Renal Effects and Underlying Molecular Mechanisms of Long-Term Salt Content Diets in Spontaneously Hypertensive Rats. PLoS ONE.

[47] Yoshida T., Kumagai H., Suzuki A., Kobayashi N., Ohkawa S., Odamaki M., Kohsaka T., Yamamoto T., Ikegaya N. (2012). Relaxin ameliorates salt-sensitive hypertension and renal fibrosis. Nephrol. Dial. Transplant. Off. Publ. Eur. Dial. Transplant. Assoc. Eur. Ren. Assoc..

[48] Yu H.C., Burrell L.M., Black M.J., Wu L.L., Dilley R.J., Cooper M.E., Johnston C.I. (1998). Salt induces myocardial and renal fibrosis in normotensive and hypertensive rats. Circulation.

[49] Hartner A., Cordasic N., Klanke B., Veelken R., Hilgers K.F. (2003). Strain differences in the development of hypertension and glomerular lesions induced by deoxycorticosterone acetate salt in mice. Nephrol. Dial. Transplant. Off. Publ. Eur. Dial. Transplant. Assoc. Eur. Ren. Assoc..

[50] Kren S., Hostetter T.H. (1999). The course of the remnant kidney model in mice. Kidney Int..

[51] Kirchhoff F., Krebs C., Abdulhag U.N., Meyer-Schwesinger C., Maas R., Helmchen U., Hilgers K.F., Wolf G., Stahl R.A., Wenzel U. (2008). Rapid development of severe end-organ damage in C57BL/6 mice by combining DOCA salt and angiotensin II. Kidney Int..

[52] Wesseling S., Ishola D.A., Joles J.A., Bluyssen H.A., Koomans H.A., Braam B. (2005). Resistance to oxidative stress by chronic infusion of angiotensin II in mouse kidney is not mediated by the AT2 receptor. Am. J. Physiol. Renal Physiol..

[53] Mikolajczyk T.P., Nosalski R., Szczepaniak P., Budzyn K., Osmenda G., Skiba D., Sagan A., Wu J., Vinh A., Marvar P.J. (2016). Role of chemokine RANTES in the regulation of perivascular inflammation, T-cell accumulation, and vascular dysfunction in hypertension. Faseb. J..

[54] Laffer C.L., Scott R.C., Titze J.M., Luft F.C., Elijovich F. (2016). Hemodynamics and Salt-and-Water Balance Link Sodium Storage and Vascular Dysfunction in Salt-Sensitive Subjects. Hypertension.

[55] Saleh M.A., McMaster W.G., Wu J., Norlander A.E., Funt S.A., Thabet S.R., Kirabo A., Xiao L., Chen W., Itani H.A. (2015). Lymphocyte adaptor protein LNK deficiency exacerbates hypertension and end-organ inflammation. J. Clin. Investig..

[56] Santisteban M.M., Ahmari N., Carvajal J.M., Zingler M.B., Qi Y., Kim S., Joseph J., Garcia-Pereira F., Johnson R.D., Shenoy V. (2015). Involvement of bone marrow cells and neuroinflammation in hypertension. Circ. Res..

[57] Sercan Alp Ö., Durlanik S., Schulz D., McGrath M., Grün J.R., Bardua M., Ikuta K., Sgouroudis E., Riedel R., Zehentmeier S. (2015). Memory CD8+ T cells colocalize with IL-7+ stromal cells in bone marrow and rest in terms of proliferation and transcription. Eur. J. Immunol..

[58] Kopp C., Linz P., Dahlmann A., Hammon M., Jantsch J., Müller D.N., Schmieder R.E., Cavallaro A., Eckardt K.U., Uder M. (2013). 23Na magnetic resonance imaging-determined tissue sodium in healthy subjects and hypertensive patients. Hypertension.

[59] Titze J. (2014). Sodium balance is not just a renal affair. Curr. Opin. Nephrol. Hypertens..

[60] Shapiro L., Dinarello C.A. (1995). Osmotic regulation of cytokine synthesis in vitro. Proc. Natl. Acad. Sci. USA.

[61] Pang D.J., Neves J.F., Sumaria N., Pennington D.J. (2012). Understanding the complexity of γδ T-cell subsets in mouse and human. Immunology.

[62] Garcia A.G., Wilson R.M., Heo J., Murthy N.R., Baid S., Ouchi N., Sam F. (2012). Interferon-γ ablation exacerbates myocardial hypertrophy in diastolic heart failure. Am. J. Physiol. Heart C.

[63] Leavy O. (2013). T cells: Salt promotes pathogenic TH17 cells. Nat. Rev. Immunol..

[64] Nguyen H., Chiasson V.L., Chatterjee P., Kopriva S.E., Young K.J., Mitchell B.M. (2013). Interleukin-17 causes Rho-kinase-mediated endothelial dysfunction and hypertension. Cardiovasc. Res..

[65] Small H.Y., Migliarino S., Czesnikiewicz-Guzik M., Guzik T.J. (2018). Hypertension: Focus on autoimmunity and oxidative stress. Free. Radic. Biol. Med..

[66] Wu J., Thabet S.R., Kirabo A., Trott D.W., Saleh M.A., Xiao L., Madhur M.S., Chen W., Harrison D.G. (2014). Inflammation and mechanical stretch promote aortic stiffening in hypertension through activation of p38 mitogen-activated protein kinase. Circ. Res..

[67] Yan G., You B., Chen S.-P., Liao J.K., Sun J. (2008). Tumor necrosis factor-α downregulates endothelial nitric oxide synthase mRNA stability via translation elongation factor 1-α 1. Circ. Res..

[68] Yun C.C., Chen Y., Lang F. (2002). Glucocorticoid activation of Na^+^/H^+^ exchanger isoform 3 revisited. The roles of SGK1 and NHERF2. J. Biol. Chem..

[69] Shibata S., Nagase M., Yoshida S., Kawachi H., Fujita T. (2007). Podocyte as the target for aldosterone: Roles of oxidative stress and Sgk1. Hypertension.

[70] Terada Y., Kuwana H., Kobayashi T., Okado T., Suzuki N., Yoshimoto T., Hirata Y., Sasaki S. (2008). Aldosterone-stimulated SGK1 activity mediates profibrotic signaling in the mesangium. J. Am. Soc. Nephrol. JASN.

[71] DiBona G.F., Esler M. (2010). Translational medicine: The antihypertensive effect of renal denervation. Am. J. Physiol. Regul. Integr. Comp. Physiol..

[72] Cheng M.H., Anderson M.S. (2012). Monogenic autoimmunity. Annu. Rev. Immunol..

[73] Vantourout P., Hayday A. (2013). Six-of-the-best: Unique contributions of γδ T cells to immunology. Nat. Rev. Immunol..

[74] Faulkner J.L., Kennard S., Huby A.C., Antonova G., Lu Q., Jaffe I.Z., Patel V.S., Fulton D.J.R., Belin de Chantemèle E.J. (2019). Progesterone Predisposes Females to Obesity-Associated Leptin-Mediated Endothelial Dysfunction via Upregulating Endothelial MR (Mineralocorticoid Receptor) Expression. Hypertension.

[75] Grabek A., Dolfi B., Klein B., Jian-Motamedi F., Chaboissier M.C., Schedl A. (2019). The Adult Adrenal Cortex Undergoes Rapid Tissue Renewal in a Sex-Specific Manner. Cell Stem Cell.

[76] Elliott P., Dyer A., Stamler R. (1989). The INTERSALT study: Results for 24-h sodium and potassium, by age and sex. INTERSALT Co-operative Research Group. J. Hum. Hypertens..

[77] Shukri M.Z., Tan J.W., Manosroi W., Pojoga L.H., Rivera A., Williams J.S., Seely E.W., Adler G.K., Jaffe I.Z., Karas R.H. (2018). Biological sex modulates the adrenal and blood pressure responses to angiotensin II. Hypertension.

[78] Chen J. (2010). Sodium sensitivity of blood pressure in Chinese populations. Curr. Hypertens. Rep..

[79] Faulkner J.L., Harwood D., Bender L., Shrestha L., Brands M.W., Morwitzer M.J., Kennard S., Antonova G., Belin de Chantemèle E.J. (2018). Lack of Suppression of Aldosterone Production Leads to Salt-Sensitive Hypertension in Female but Not Male Balb/C Mice. Hypertension.

[80] Olivieri O., Pizzolo F., Ciacciarelli A., Corrocher R., Signorelli D., Falcone S., Blengio G.S. (2008). Menopause not aldosterone-to-renin ratio predicts blood pressure response to a mineralocorticoid receptor antagonist in primary care hypertensive patients. Am. J. Hypertens..

[81] Gwoo S., Kim Y.N., Shin H.S., Jung Y.S., Rim H. (2014). Predictors of hyperkalemia risk after hypertension control with aldosterone blockade according to the presence or absence of chronic kidney disease. Nephron Clin. Pract..

[82] Whelton P.K., He J., Appel L.J., Cutler J.A., Havas S., Kotchen T.A., Roccella E.J., Stout R., Vallbona C., Winston M.C. (2002). Primary prevention of hypertension: Clinical and public health advisory from The National High Blood Pressure Education Program. JAMA.

[83] Kuro-o M., Matsumura Y., Aizawa H., Kawaguchi H., Suga T., Utsugi T., Ohyama Y., Kurabayashi M., Kaname T., Kume E. (1997). Mutation of the mouse klotho gene leads to a syndrome resembling ageing. Nature.

[84] Ong K.L., Cheung B.M., Man Y.B., Lau C.P., Lam K.S. (2007). Prevalence, awareness, treatment, and control of hypertension among United States adults 1999–2004. Hypertension.

[85] Ishibashi K., Oshima T., Matsuura H., Watanabe M., Ishida M., Ishida T., Ozono R., Kajiyama G., Kanbe M. (1994). Effects of age and sex on sodium chloride sensitivity: Association with plasma renin activity. Clin. Nephrol..

[86] Weinberger M.H., Miller J.Z., Luft F.C., Grim C.E., Fineberg N.S. (1986). Definitions and characteristics of sodium sensitivity and blood pressure resistance. Hypertension.

[87] De Moudt S., Hendrickx J.O., Neutel C., De Munck D., Leloup A., De Meyer G.R.Y., Martinet W., Fransen P. (2022). Progressive aortic stiffness in aging C57Bl/6 mice displays altered contractile behaviour and extracellular matrix changes. Commun. Biol..

[88] Wirth A., Wang S., Takefuji M., Tang C., Althoff T.F., Schweda F., Wettschureck N., Offermanns S. (2016). Age-dependent blood pressure elevation is due to increased vascular smooth muscle tone mediated by G-protein signalling. Cardiovasc. Res..

[89] Zhou X., Chen K., Lei H., Sun Z. (2015). Klotho gene deficiency causes salt-sensitive hypertension via monocyte chemotactic protein-1/CC chemokine receptor 2-mediated inflammation. J. Am. Soc. Nephrol. JASN.

[90] Takenaka T., Inoue T., Miyazaki T., Kobori H., Nishiyama A., Ishii N., Hayashi M., Suzuki H. (2018). Klotho Ameliorates Medullary Fibrosis and Pressure Natriuresis in Hypertensive Rat Kidneys. Hypertension.

[91] Yamazaki Y., Imura A., Urakawa I., Shimada T., Murakami J., Aono Y., Hasegawa H., Yamashita T., Nakatani K., Saito Y. (2010). Establishment of sandwich ELISA for soluble alpha-Klotho measurement: Age-dependent change of soluble alpha-Klotho levels in healthy subjects. Biochem. Biophys. Res. Commun..

[92] Zhou X., Wang X. (2015). Klotho: A novel biomarker for cancer. J. Cancer Res. Clin. Oncol..

[93] Chen K., Sun Z. (2018). Activation of DNA demethylases attenuates aging-associated arterial stiffening and hypertension. Aging Cell.

[94] Kawarazaki W., Mizuno R., Nishimoto M., Ayuzawa N., Hirohama D., Ueda K., Kawakami-Mori F., Oba S., Marumo T., Fujita T. (2020). Salt causes aging-associated hypertension via vascular Wnt5a under Klotho deficiency. J. Clin. Investig..

[95] Trott D.W., Thabet S.R., Kirabo A., Saleh M.A., Itani H., Norlander A.E., Wu J., Goldstein A., Arendshorst W.J., Madhur M.S. (2014). Oligoclonal CD8+ T cells play a critical role in the development of hypertension. Hypertension.

[96] Fejes-Tóth G., Frindt G., Náray-Fejes-Tóth A., Palmer L.G. (2008). Epithelial Na+ channel activation and processing in mice lacking SGK1. Am. J. Physiol. Renal Physiol..

[97] Muntner P., Carey R.M., Gidding S., Jones D.W., Taler S.J., Wright J.T., Whelton P.K. (2018). Potential US Population Impact of the 2017 ACC/AHA High Blood Pressure Guideline. Circulation.

[98] Bridges L.E., Williams C.L., Pointer M.A., Awumey E.M. (2011). Mesenteric artery contraction and relaxation studies using automated wire myography. J. Vis. Exp. JoVE.

[99] Rangan G.K., Tesch G.H. (2007). Quantification of renal pathology by image analysis. Nephrology.

