# Supplementary material for: Dietary High Salt Intake Exacerbates SGK1-Mediated T Cell Pathogenicity in L-NAME/High Salt-Induced Hypertension
Source: Int J Mol Sci. 2024 Apr 16;25(8):4402. doi: 10.3390/ijms25084402 (PMC11050194; doi:10.3390/ijms25084402)
Supplement: Supplementary file 1 [file ijms-25-04402-s001.zip › ijms-2932692-supplementary.pdf]

SUPPLEMENTARY FIGURES

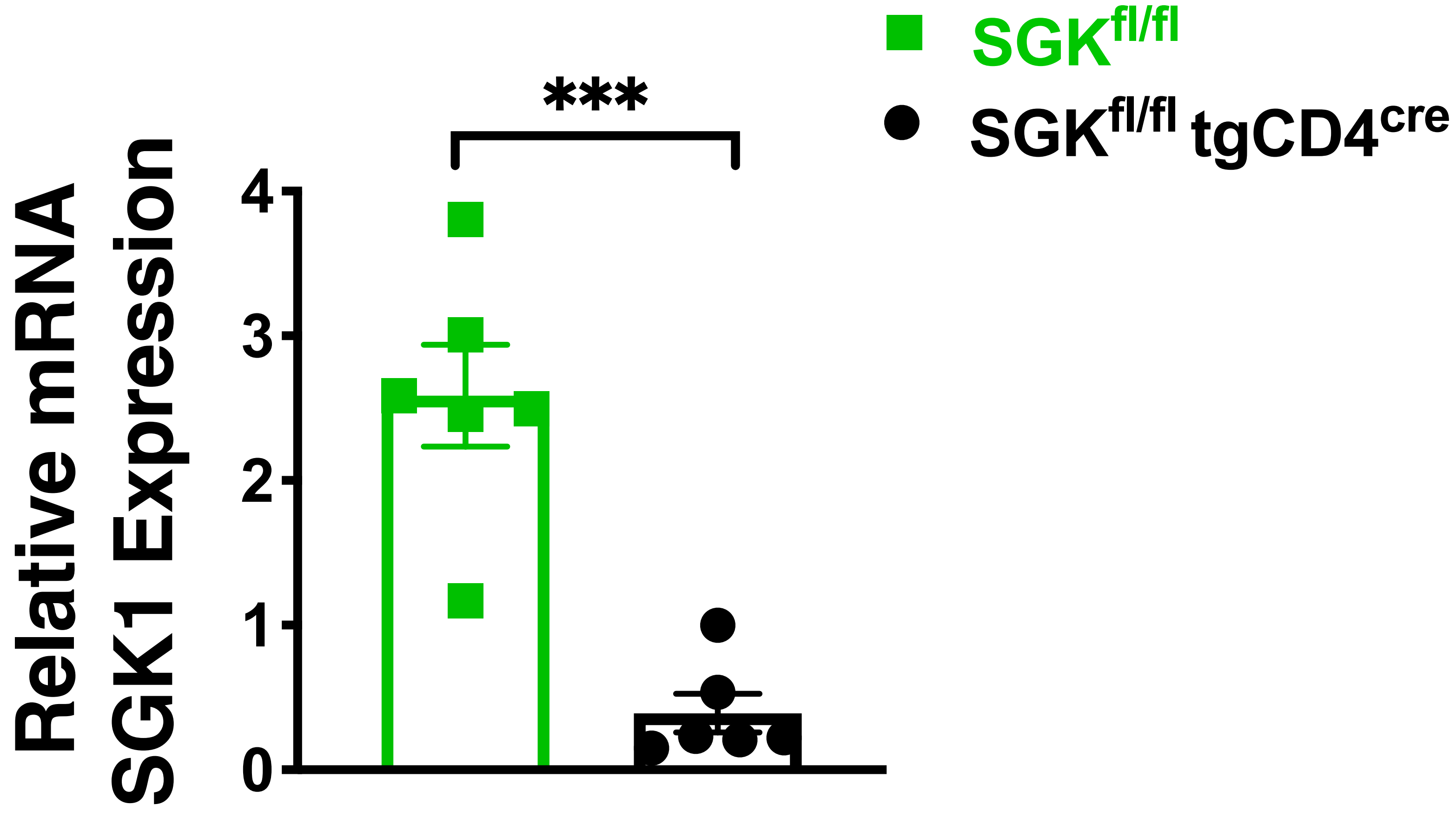

Figure S1. SGK1 expression in bone marrow T cells.

T cells were isolated from the bone marrow of SGK1$^{fl/fl}$ and SGK1$^{fl/fl}$tgCD4^{cre} mice using a Miltenyi cell separation kit according to the manufacturer's instructions and a MACS magnetic cell sorter. SGK1 expression was then quantified by RT-qPCR (N=6). Data are expressed as mean ± SEM, P-value calculated by independent t-test, *** $P < 0.001$.

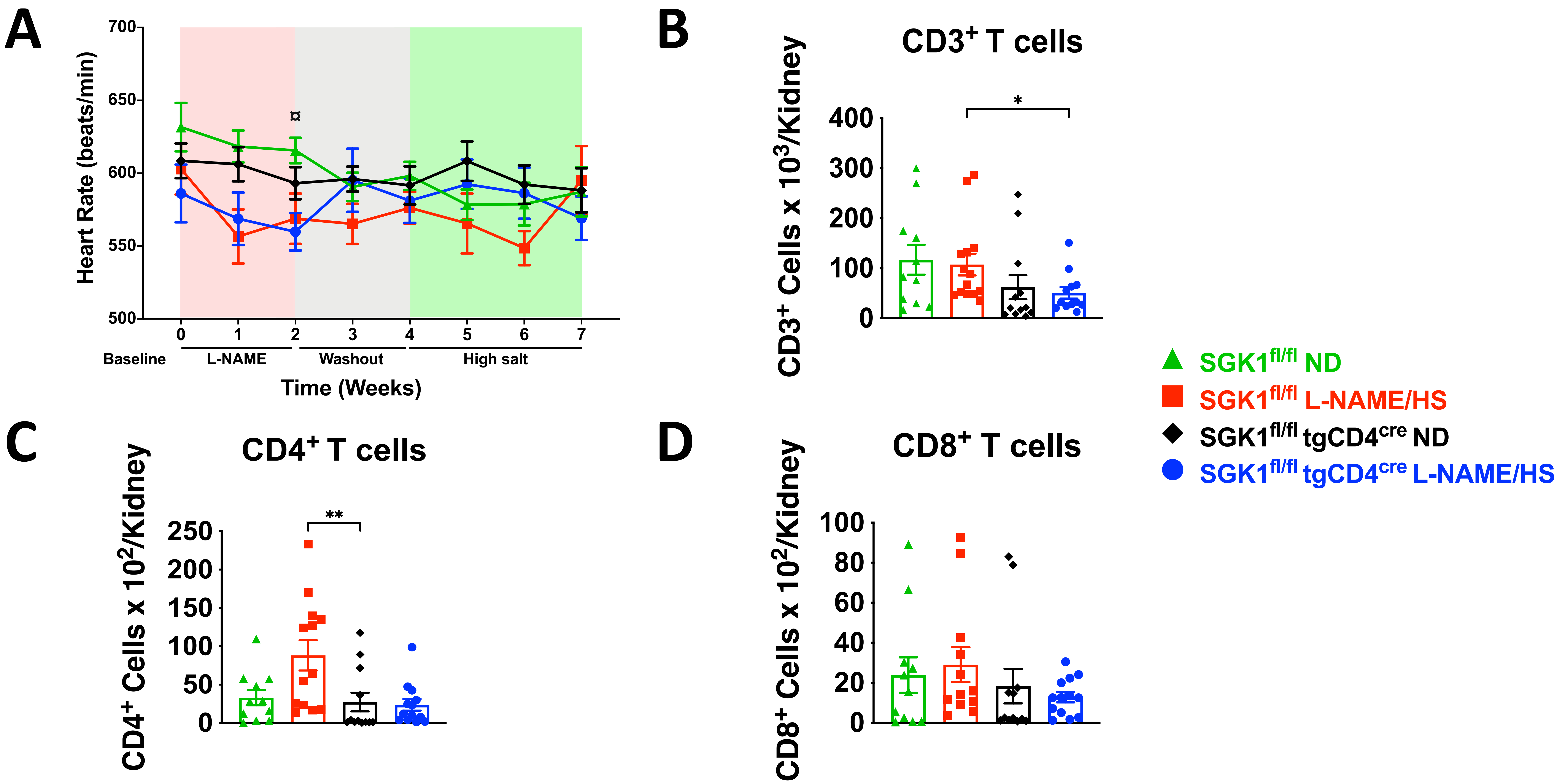

Figure S2. Differences in Heart rate and in total renal CD3^{+}, CD4^{+} and CD8^{+} T lymphocytes.
(A) Heart rate measured noninvasively using the tail-cuff method (N=12 -15). Data are expressed as mean ± SEM, and P-values calculated by multiple t-tests at each time point multiplied by the number of comparisons are shown. ¤ P< 0.05 for SGK1$^{fl/fl}$tgCD4^{Cre} L-NAME/HS vs. SGK1$^{fl/fl}$tg^{CD4cre} ND mice; **(B)** CD3^{+} T lymphocytes; **(C)** CD4^{+} T lymphocytes; **(D)** CD8^{+} T lymphocytes (N=11-14). Data are expressed as mean ± SEM, and P-values calculated by 2-way ANOVA are shown. *P<0.05, ** P< 0.01.
